# Baicalein attenuates OVA-induced allergic airway inflammation through the inhibition of the NF-κB signaling pathway

**DOI:** 10.18632/aging.102371

**Published:** 2019-11-06

**Authors:** Tingting Xu, Xiangting Ge, Chun Lu, Wei Dai, Hongjin Chen, Zhongxiang Xiao, Liqin Wu, Guang Liang, Songmin Ying, Yali Zhang, Yuanrong Dai

**Affiliations:** 1 The Second Affiliated Hospital and Yuying Children’s Hospital of Wenzhou Medical University, Wenzhou, China; 2 Chemical Biology Research Center, School of Pharmaceutical Sciences, Wenzhou Medical University, Wenzhou, China; 3 Affiliated Yueqing Hospital, Wenzhou Medical University, Wenzhou, China; 4 Department of Pharmacology and Key Laboratory of Respiratory Disease of Zhejiang Province, Department of Respiratory and Critical Care Medicine, Second Affiliated Hospital, Institute of Respiratory Diseases, Zhejiang University School of Medicine, Hangzhou, China

**Keywords:** Baicalein, asthma, inflammation, NF-κB, iNOS

## Abstract

Asthma is a type of chronic lung inflammation with restrictions in effective therapy. NF-κB pathway activation has been suggested to play an important role in the pathogenesis of asthma. Baicalein, one of the major active flavonoids found in *Scutellaria baicalensis*, exhibits potent anti-inflammatory properties by inhibiting NF-κB activity. Herein, we report that Baicalein significantly reduces OVA-induced airway hyperresponsiveness (AHR), airway inflammation, serum IgE levels, mucus production, and collagen deposition around the airway. Additionally, western blot analysis and immunofluorescence assay showed that Baicalein attenuates the activation of NF-κB, which was mainly reflected by IκBα phosphorylation and degradation, p65 nuclear translocation and downstream iNOS expression. Furthermore, in human epithelial cells, Baicalein blocked TNF-α-induced NF-κB activation. Our study provides evidence that Baicalein administration alleviates the pathological changes in asthma through inactivating the NF-κB/iNOS pathway. Baicalein might be a promising potential therapy agent for patients with allergic asthma in the future.

## INTRODUCTION

Asthma is a common and heterogeneous chronic respiratory disease characterized by variable symptoms of wheeze, shortness of breath, chest tightness and/or cough, and variable expiratory airflow limitation [1]. According to epidemiological investigations, asthma affects approximately 1–18% of the population in different countries.

Type 2 inflammation is an important molecular mechanism in asthma. Recently, T helper type 2 (Th2) cells and type 2 innate lymphoid cells (ILC2s) were recognized as important cells involved in allergic eosinophilic asthma [2, 3]. These two types of cells contribute to increases in eosinophilic inflammation, immunoglobulin E (IgE) production, airway hyperresponsiveness (AHR), and mucus hypersecretion through the production of Th2 cytokines (interleukins 4, 5, and 13) [4, 5].

The long-term goals for asthma management are to achieve good symptom control, maintain normal activity levels, reduce the future risk of exacerbations, prevent fixed airflow limitation and minimize side effects. Currently, control-based asthma management consists of nonpharmacological approaches, such as allergen avoidance, and pharmacological approaches. The mainstay of pharmacological approaches to treat asthma is daily inhaled corticosteroids (ICS) combined together with long-acting β2 agonists (LABA). Although great achievements in pharmacological approaches to treat asthma have been made, some patients (approximately 10 to 25%) remain symptomatic after undergoing the optimal ICS + LABA therapy [6]. In addition, corticosteroids do not function directly on pulmonary structural changes, nor are they sufficient to suppress IL-13-induced mucus dysfunction [7]. Furthermore, as corticosteroids may contribute to pneumonia, hypertension, hyperlipidemia, peptic ulcers, myopathy, cataracts and growth inhibition in children during the first year of treatment, their side effects cannot be ignored [8].

Except for the treatment regime mentioned above, the widespread application of complementary or alternative medicine (CAM) in patients with asthma has increased the demand for research on its use in asthma. CAM in asthma treatment consists of acupuncture, herbal medicine, yoga, breathing exercises, relaxation therapies, and nutritional therapies, among others [9]. Among these, herbal medicine is the most popular CAM in asthma treatment. A study reported that 11–40% of people with asthma are inclined to use herbal remedies [10].

In China, many herbs have long been used to treat asthma and airway inflammation. Furthermore, substantial evidence has shown the efficacy and safety of many traditional Chinese medicines (TCMs), such as Mai-men-dong-tang and Dingchuan-tang [11], in patients with asthma in China and in many other countries [12, 13]. *Boswellia*, an herb used in Ayurvedic medicine (a traditional Indian system of healthcare), had a beneficial effect on patients with bronchial asthma in a clinical trial [14, 15]. Several studies have revealed that Pycnogenol, a standardized extract from French maritime pine bark, improves lung function and reduces symptoms in patients with asthma [16, 17]. Furthermore, many important drugs currently used in the treatment of asthma originated from herbs. For example, the traditional Chinese remedy ‘ma huang’ is the herbal origin of ephedrine, and theophylline was developed from ma huang tea leaves.

Baicalein (5,6,7-trihydroxy-2-phenyl-4H-1-benzopyran-4-one) is one of the major flavonoids derived from the root of *Scutellaria baicalensis*, namely, the traditional Chinese medicinal herb Huang Qin [18]. Baicalein possesses multiple pharmacological properties in various diseases, including cardiovascular diseases [19], hypertension [20], bacterial infection [21] and cancer [22]. Meanwhile, accumulating evidence has reported the antiallergic effects of Baicalein, but the mechanisms of these effects remain unknown. Baicalein inhibited cigarette smoke extract (CSE)-induced inflammatory cytokine production through the inactivation of NF-κB in human mast cells [23]. NF-κB is a pleiotropic transcription factor, and its roles in the pathogenesis of asthma have been explored in mouse models of allergic airway inflammation and in human patients with asthma [24]. CC10-IκBαSR transgenic mice, which are refractory to IκBα degradation and NF-κB activation in the lung epithelium, were demonstrated to be strongly protected from airway inflammation induced by ovalbumin (OVA) [25, 26]. Furthermore, NF-κB–specific decoy oligonucleotide and p65-specific antisense oligonucleotides were reported to have beneficial effects in experimental asthma models [27]. In this study, we elucidate whether Baicalein mitigates OVA-induced allergy airway inflammation through regulating the NF-κB pathway both *in vitro* and *in vivo*.

## RESULTS

### Baicalein relieves OVA-induced AHR in mice

**Figure 1:**
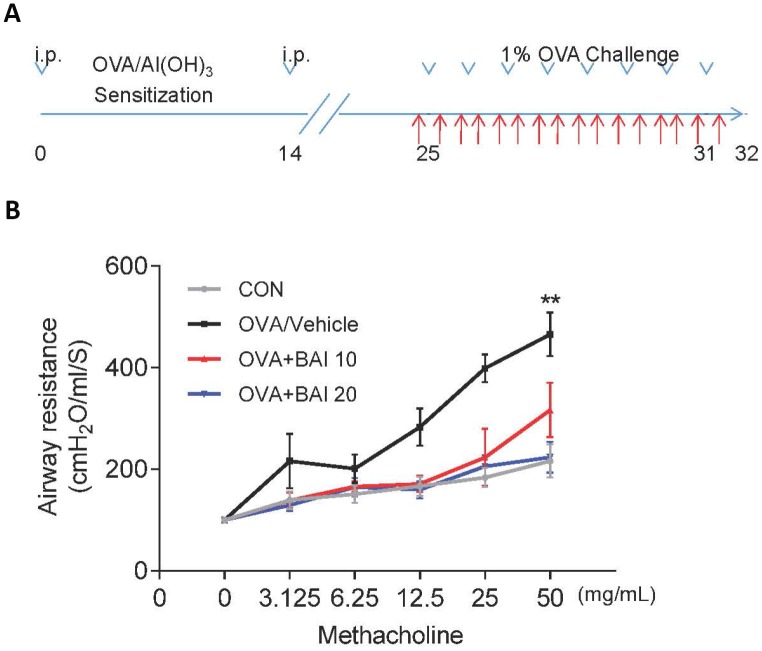
**Baicalein relieves OVA-induced AHR in mice.** (**A**) The construction of a model of OVA-induced allergic airway inflammation. Mice were sensitized by OVA/Al(OH)_3_ on day 0 and day 14, while from days 25 to 31, the mice were exposed to 1% OVA aerosol for 7 consecutive days. (**B**) Airway responsiveness was assessed as the mean response of mechanically ventilated mice to increased doses of Mch (mean ± SEM; n = 6 per group; ^**^P < 0.01 compared with the control group).

AHR is generally used to describe increased airway smooth muscle contraction that contributes to obstruction in people with asthma, which is a form of inflammation [28]. In laboratories studying lung function, AHR is most frequently established with inhaled Methacholine (Mch) rather than histamine, osmotic agents, exercise, or eucapnic voluntary hyperventilation. Therefore, airway resistance (Rn) in mechanically ventilated mice in response to increasing concentrations of Mch was measured to determine whether Baicalein impacts AHR. As predicted, OVA sensitization and challenge led to an AHR, which is typically reflected by high Rn (Figure 1B). However, the Rn of allergic mice that were treated with Baicalein (10 mg/kg and 20 mg/kg) was significantly reduced in a dose-dependent manner relative to that in OVA-sensitized and challenged mice.

### Baicalein reduces OVA-induced serum IgE and Th2 cytokine levels

**Figure 2:**
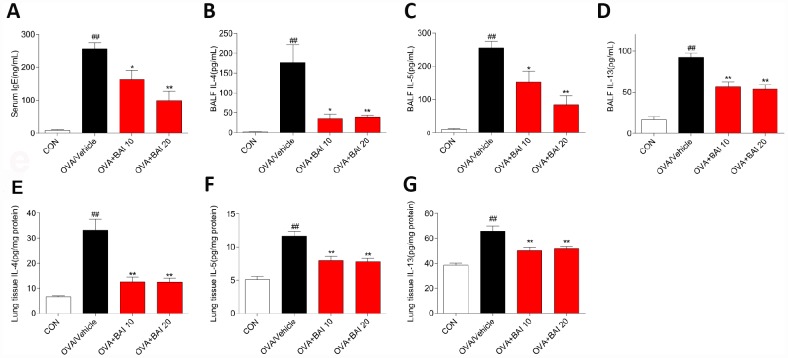
**Baicalein reduces OVA-induced Th2 inflammation.** The OVA/Al(OH)_3_ model is characterized by Th2-driven airway inflammation. To determine the effect of Baicalein on Th2 airway inflammation, ELISA was performed to detect the levels of IgE in serum (**A**) and IL-4, IL-5, and IL-13 in BALF (**B**–**D**) and lung homogenate (**E**–**G**) (results are presented as the mean ± SEM. n = 6 mice per group; ^##^*P* < 0.01 compared with the control group; *P < 0.05, **P < 0.01 compared with the OVA/Vehicle group).

**Figure 3:**
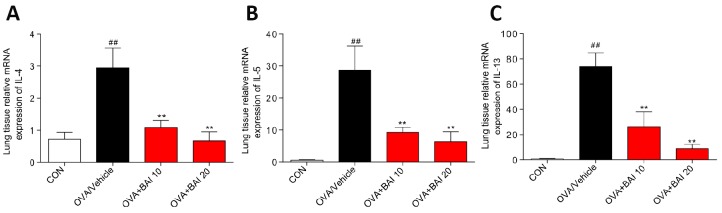
**Baicalein inhibits OVA-induced IL-4, IL-5, and IL-13 expression at the mRNA level.** The mRNA levels of IL-4 (**A**), IL-5 (**B**), and IL-13 (**C**) were determined by using RT-qPCR and were normalized to those of β-actin. (Results are presented as the mean ± SEM; n = 6 mice per group. *^##^P* < 0.01 vs the control group; *P < 0.05, **P < 0.01 vs the OVA/Vehicle group).

Serum levels of total IgE were determined by enzyme linked immunosorbent assay (ELISA) to evaluate the effect of Baicalein on the OVA-specific Th2 response in vivo. The serum total IgE level was markedly increased in OVA-challenged mice, and Baicalein treatment suppressed total IgE production in asthmatic mice even at a low dose (10 mg/kg, Figure 2A). After inhalation of OVA, the sensitized mice exhibited significantly increased levels of released Th2 cytokines, (IL-4, IL-5 and IL-13) in bronchoalveolar lavage fluid (BALF) and lung tissue compared to those observed in the saline-treated control mice. As shown in Figure 2B–2G, the OVA-induced increases in these cytokines in both BALF (Figure 2B–2D) and lung tissues (Figure 2E–2G) were significantly reduced by the administration of Baicalein. We further explored the effect of Baicalein on the Th2 response by assessing the mRNA expression levels of these cytokines. As shown in Figure 3A–3C, the administration of Baicalein relieved the OVA-induced increase in IL-4, IL-5, and IL-13 mRNA expression levels.

### Baicalein suppresses OVA-induced inflammatory cell recruitment

**Figure 4:**
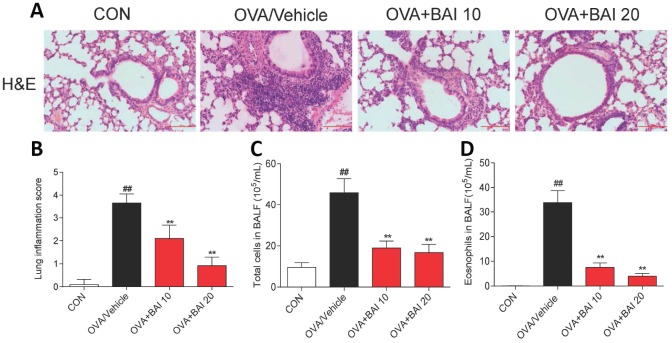
**Baicalein suppresses OVA-induced inflammatory cell recruitment.** (**A**) Histologic lung sections were stained with H&E, which showed that Baicalein reduces inflammatory cell recruitment and infiltration into the airway. Image are shown at 200× magnification with a scale bar representing 100 μm. (**B**) Lung inflammatory scores were assessed by histological analysis of lung tissues. Baicalein reduced the numbers of total cells (**C**) and eosinophils (**D**) in BALF following OVA challenge (Results are presented as the mean ± SEM. n = 6 mice per group; *^##^P* < 0.01 compared with the control group; *P < 0.05, **P < 0.01 compared with the OVA/Vehicle group).

To further determine the effect of Baicalein on OVA-induced airway inflammation, hematoxylin and eosin (H&E) staining was conducted. As shown in Figure 4A and 4B, Baicalein markedly relieved the infiltration of inflammatory cells into the peribronchiolar and perivascular connective tissues. Furthermore, asthmatic mice after OVA inhalation presented thickened airway walls and confined lumens and shed tracheal epithelial cells, suggesting that Baicalein treatment relieves these pathologic changes.

BALF was collected 24 h after the last OVA aerosol challenge, and the total and differential cell counts were determined. OVA challenge significantly increased the total cell (Figure 4C) and eosinophil counts (Figure 4D) in BALF compared to those in control mice. The oral administration of Baicalein drastically decreased the total cell and eosinophil counts compared to those in the saline-administered control mice.

### Baicalein attenuates OVA-induced mucus production

**Figure 5:**
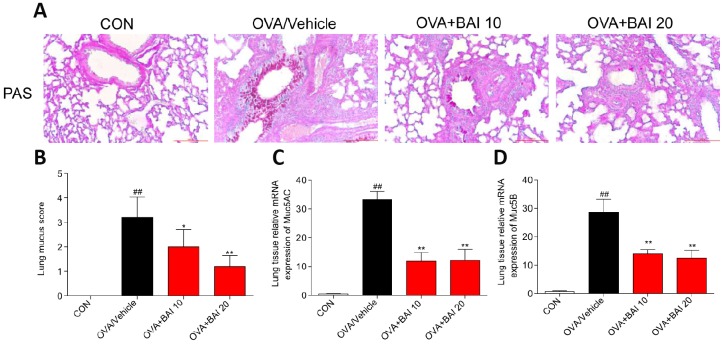
**Baicalein attenuates OVA-induced mucus production.** Goblet cell hyperplasia and mucin gene expression were used to measure mucus production in mice. (**A**) PAS staining was performed to identify goblet cell hyperplasia in the airway epithelium. Images are shown at 200× magnification with a scale bar representing 100 μm. (**B**) Quantification of mucus-producing goblet cells in lung tissues detected by PAS staining. The mRNA levels of the mucus-related genes MUC5AC (**C**) and MUC5B (**D**) were quantified by RT-qPCR and normalized to those of β-actin (n = 6 mice per group; *^##^P* < 0.01 compared with the control group; *P < 0.05 compared with OVA/Vehicle group, **P < 0.01 compared with the OVA/Vehicle group).

The formation of mucus in small and large bronchioles is an important aspect of allergic lung inflammation, and goblet cell hyperplasia and submucosal gland hypertrophy in asthmatic airways can be seen even in some patients with newly diagnosed asthma [28]. As visualized by Periodic Acid Schiff (PAS) staining, OVA exposure increased mucus production by airway epithelial cells (Figure 5A–5B). However, Baicalein treatment significantly decreased the production and secretion of mucus. In addition, we determined the expression of the mucus secretion-related genes MUC5AC and MUC5B. In accordance with the results of PAS staining, Baicalein markedly reduced the expression levels of MUC5AC (Figure 5C) and MUC5B (Figure 5D).

### Baicalein suppresses continuous OVA challenge induced collagen deposition

**Figure 6:**
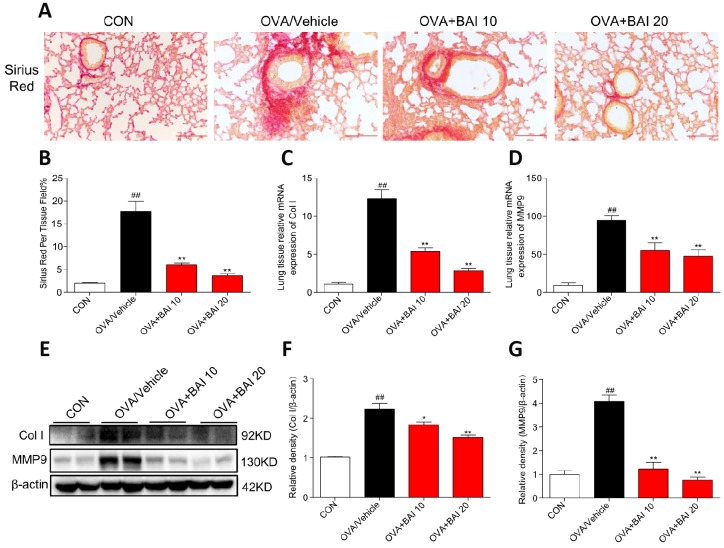
**Baicalein suppresses continuously OVA challenge induced collagen deposition.** (**A**) Lung tissue sections were stained with Sirius Red to assess collagen deposition. Images are shown at 200× magnification with a scale bar representing 100 μm. (**B**) A bar graph showing quantified collagen deposition areas (%) detected by Sirius Red staining. (**C**–**D**) Expression levels of collagen I (Col I) and MMP9 in the lung tissues of mice in each group were determined by RT-qPCR. Lung tissues from each group were extracted for western blotting to analyze collagen I and MMP-9 expression, with β-actin used as a loading control. Proteins from three mouse lung tissues were pooled together, n = 6 in one group (**F**–**G**). The results are presented as the mean ± SEM; *^##^P* < 0.01 compared with the control group; *P < 0.05, **P < 0.01 compared with the OVA/Vehicle group.

The extent of collagen deposition was evaluated by Sirius Red staining. As shown in Figure 6A and 6B, marked collagen deposition over the interstitium of the airways was observed after OVA challenge. However, these increases in airway collagen deposition and fibrosis were reversed by Baicalein administration. Metalloproteinase-9 (MMP-9) is thought to be involved in collagen deposition in airway walls, which contributes to narrowed airways [29]. To further verify the role of MMP-9 in collagen deposition, we determined MMP-9 and collagen I expression at the mRNA and protein levels. The MMP-9 and collagen I expression levels in lung tissue were significantly elevated in asthmatic mice compared with those in the control mice (Figure 6C–6G), whereas these elevations in expression were abolished by Baicalein at both doses (10 mg/kg and 20 mg/kg). Our results indicate that Baicalein restrained OVA-induced MMP-9 and collagen I expression, further contributing to the suppression of extracellular matrix (ECM) deposition and fibrosis.

### Baicalein inhibits OVA-induced NF-κB activation and downstream iNOS expression in allergic airway inflammation

**Figure 7:**
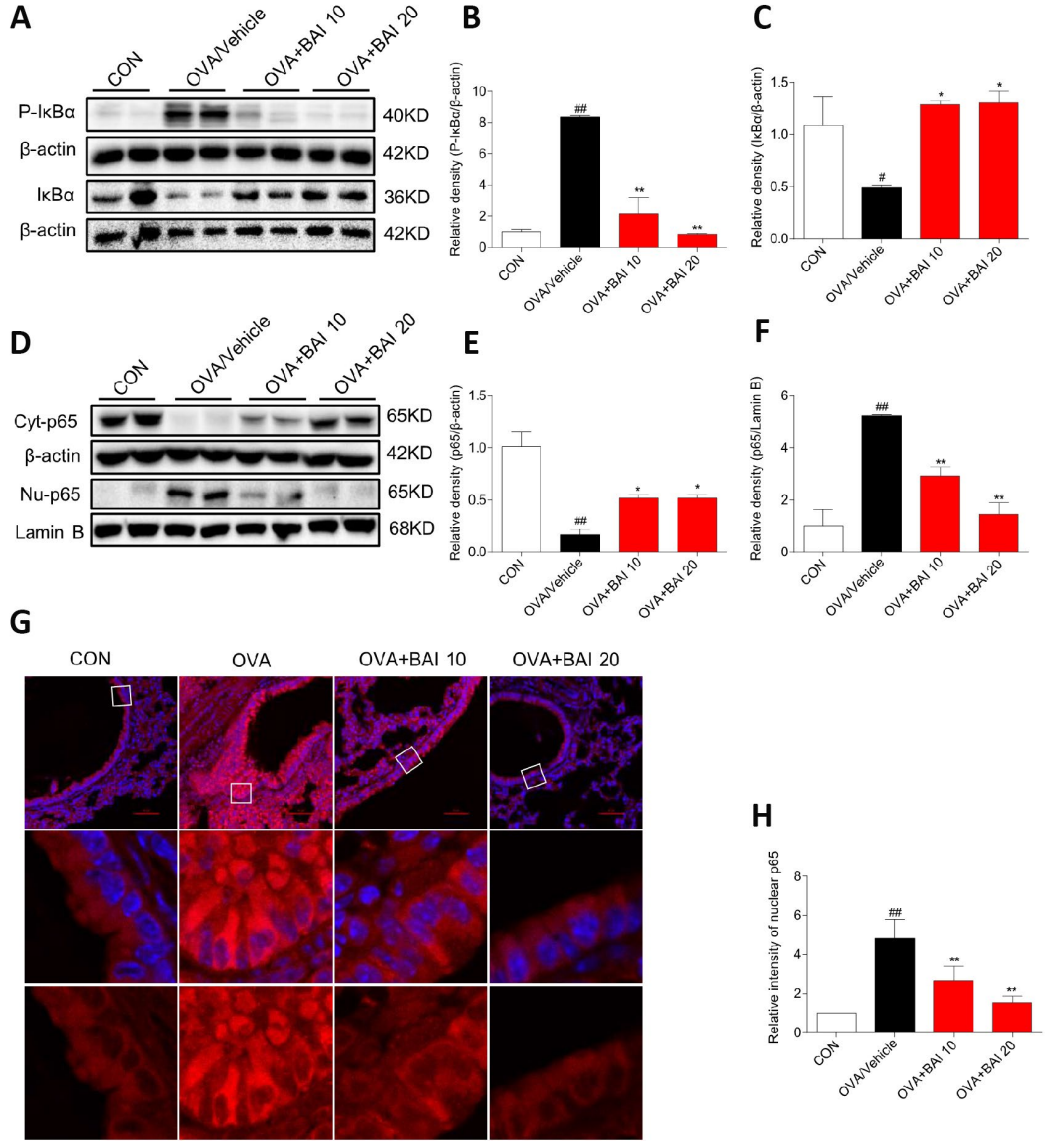
**Baicalein inhibits OVA-induced NF-κB pathway activation.** (**A**) The protein levels of P-IκBα and IκBα in the lung tissues of mice in each group were examined by western blot analysis with β-actin used as an internal control. (**B**–**C**) A bar graph shows the quantification of P-IκBα, IκBα and β-actin by densitometry. (**D**–**F**) Cytosolic (upper blot) and nuclear (lower blot) p65 levels were determined by western blot. β-actin and Lamin B were used as loading controls. Proteins from three mouse lung tissues were pooled together. n = 6 in one group. (**G**) Immunofluorescence staining for p65 (red, Cy3) in lung tissues of mice at 100× magnification. Nuclei were stained with DAPI (blue). (**H**) Relative nuclear immunostaining intensity of p65 was quantified. The results are presented as the mean ± SEM. n = 6 mice per group; *^##^P < 0.01* compared with the control group; *P < 0.05, **P < 0.01 compared with the OVA/Vehicle group.

**Figure 8:**
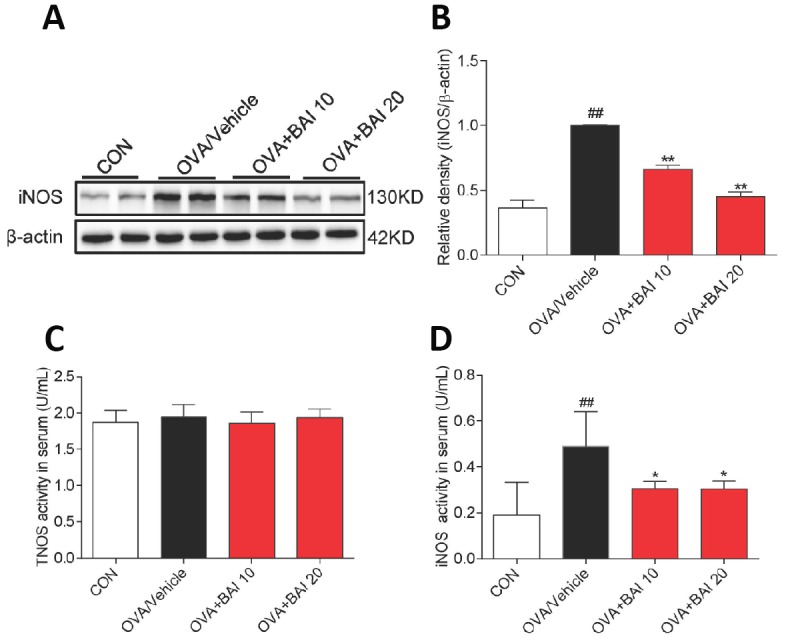
**iNOS expression and activity are suppressed upon Baicalein treatment.** (**A**) The expression of iNOS in the total proteins of lung tissues from mice in each group was detected by western blot analysis, and β-actin was used as an internal control. Proteins from three mouse lung tissues were pooled together. n = 6 in one group. (**B**) A bar graph shows the quantification of iNOS and β-actin by densitometry. Total NO synthase (TNOS, **C**) and inducible NO synthase (iNOS, **D**) activities in mouse serum were measured using an NOS Assay Kit (Error bars represent the mean ± SEM; *^##^P < 0.01* compared with the control group; **P < 0.01 compared with the OVA/Vehicle group).

Next, we detected NF-κB pathway activation in lung tissues to explore the possible mechanism by which Baicalein relieves asthma. OVA-challenged mice showed markedly increased IκBα phosphorylation and IκBα degradation (Figure 7A–7C). Western blot analysis (Figure 7D–7F) and immunofluorescence assay (Figure 7G–7H) indicated that NF-κB p65 translocated from the cytosol to the nucleus in our study. However, treatment with Baicalein reversed OVA-induced NF-κB activation in a dose-dependent manner. NO derived from iNOS activation is involved in inflammatory cell recruitment and changes in lung structure [30]. In addition, the iNOS pathway is related to the modulation of NF-κB expression [31]. Based on these findings, we measured the expression of iNOS in lung homogenates. As presented in Figure 8A–8B, the expression of iNOS was 2.1-fold higher in mice exposed to OVA than in mice exposed to normal saline. The oral administration of Baicalein reduced the expression of iNOS in a concentration-dependent manner. Moreover, the activity levels of TNOS and iNOS were also measured. iNOS activity was significantly decreased after the oral administration of Baicalein (Figure 8D), but the activity of TNOS showed no significant change (Figure 8C). Thus, from the results mentioned above, we concluded that Baicalein significantly reversed OVA-induced IκBα degradation, NF-κB p65 nuclear translocation, and the expression and activity of iNOS, suggesting that Baicalein exerts its anti-allergic effect via the inhibition of NF-κB/iNOS activation.

### Baicalein inhibits TNF-α–induced NF-κB activation in BEAS-2B cells

Activation of the classical NF-κB pathway in the airway epithelium plays a critical role in allergic airway inflammation [26]. In our study, Baicalein had a significant effect on the activation of the NF-κB pathway in a murine model. To investigate the anti-inflammatory mechanisms of Baicalein in a relevant airway cell type, we studied the effects of Baicalein on TNF-α-induced activation of the NF-κB pathway in Human normal bronchial epithelial (BEAS-2B) cells.

**Figure 9:**
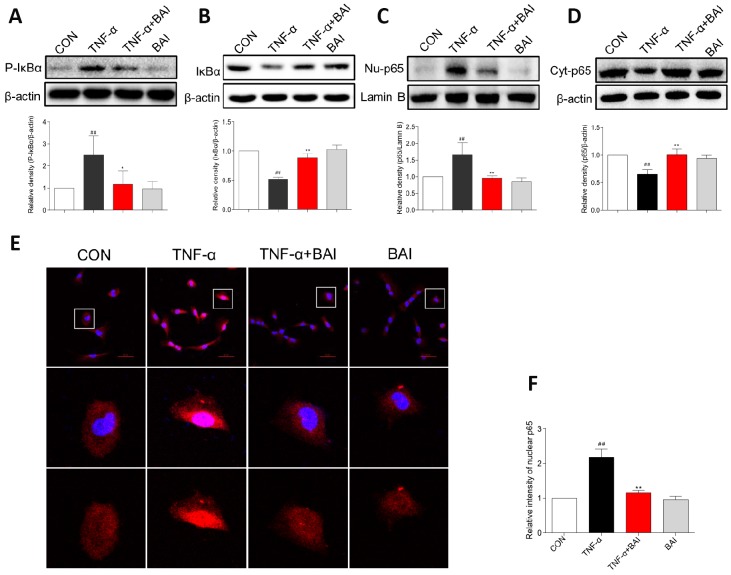
**Baicalein inhibits TNF-α-induced NF-κB activation in BEAS-2B cells.** BEAS-2B cells were pretreated with vehicle control (DMSO) or Baicalein (2.5 μM) for 30 min, followed by exposure to TNF-α (10 ng/mL) for 60 min. Total proteins were extracted and analyzed for P- IκBα (**A**) and IκBα (**B**) expression by western blot analysis, with β-actin used as the internal control. After BEAS-2B cells were exposed to TNF-α (10 ng/mL) for 2 h, the nuclear and cytosolic proteins were separated using a cytoplasmic and nuclear protein extraction kit, and the nuclear (**C**) and cytosolic (**D**) p65 levels were determined by western blot analysis. β-actin and Lamin B were used as internal controls. (**E**) p65 staining was carried out, and p65 levels were detected by Cy3-conjugated secondary antibody (red). Cells were counterstained with DAPI (blue) and are shown with a scale bar indicating 50 μm. (**F**) Relative nuclear immunostaining intensity of p65 was quantified. (mean ± SEM of more than three independent experiments; ^##^*P* < 0.01 compared with control, *P < 0.05 compared to TNF-α, **P < 0.01 compared to TNF-α).

TNF-α is also an important cytokine in patients with asthma and contributes to the inflammatory response in the asthmatic airway [32]. Additionally, several studies have supported a central role for TNF-α in the development of AHR and other features of the asthma paradigm [33]. Furthermore, as a stimulator, TNF-α activates NF-κB in lung epithelial cells [34]. In this study, TNF-α (10 ng/mL) induced the phosphorylation of IκBα and IκBα degradation after 60 min, and p65 nuclear translocation was detected after 2 h. As shown in Figure 9A–9B, TNF-α strongly enhanced the phosphorylation of IκBα and accelerated the degradation of IκBα in BEAS-2B cells. However, Baicalein (2.5 μM) noticeably blocked the changes induced by TNF-α. Western blot analysis (Figure 9C–9D) and immunofluorescence assay (Figure 9E) indicated that Baicalein significantly impeded NF-κB p65 translocation from the cytoplasm to the nucleus after exposure to TNF-α. In addition, treatment with Baicalein alone did not affect NF-κB signaling pathways.

## DISCUSSION

Asthma is ranked as the 14^th^ most important chronic disease worldwide in terms of its prevalence, and the extent and duration of disability due to asthma [35]. According to the Global Asthma Report in 2014, the latest revised global estimates of asthma suggest that more than 334 million people worldwide suffer from asthma, and the prevalence of asthma and the burden of asthma-related disability are increasing [36]. Asthma is an eosinophilic/Th2 disorder, and novel therapeutics targeting Th2 cytokines (IL-4, IL-5 and IL-13) and IgE have achieved excellent improvements in disease control, although these therapeutics are applicable to only a sub group of patients in clinical studies [5, 37]. Thus, other novel therapies are urgently needed to better treat patients at all levels.

NF-κB was previously reported to be involved in the pathogenesis of asthma, and evidence for the activation of NF-κB in bronchiolar epithelium has been observed in both animal models of allergic airway disease and patients with asthma [38]. Baicalein protects against inflammatory diseases via the inhibition of NF-κB transactivation [23, 39, 40]. To gain further insight into the mechanism by which Baicalein regulates NF-κB *in vivo*, we examined the effect of Baicalein on NF-κB signaling. As shown in Figure 7, OVA-induced NF-κB activation, which promotes IκBα phosphorylation and degradation and NF-κB nuclear translocation, was significantly blocked by treatment with Baicalein. We also detected IκBα mRNA expression levels before and after OVA/Baicalein treatment, and, as shown in Supplementary Figure 2, there was no significant difference in IκBα mRNA expression between the OVA/vehicle and control groups. In addition, treatment with Baicalein did not affect IκBα mRNA expression. This result indicates that OVA/Baicalein treatment did not affect the transcription of IκBα. Therefore, we believe that the effects of Baicalein on IκBα protein expression depend on IκBα degradation. To verify the anti-inflammatory mechanisms of Baicalein in a relevant airway cell type, we studied the effects of Baicalein on the TNF-α induced activation of the NF-κB pathway in BEAS-2B cells. Similar to the results of *in vivo* experiments, TNF-α-induced NF-κB activation was blocked by pretreatment with Baicalein. We then used Bay11-7082 (BAY), an NF-κB inhibitor, as a positive control; TNF-α-induced NF-κB activation was blocked by pretreatment with either BAY or Baicalein (Supplementary Figure 1). ROS, interleukins, and lipopolysaccharide were also involved in the activation of NF-κB pathway during the pathogenesis asthma [41]. Here, we showed that Baicalein could block the TNF-α-induced NF-κB activation. However, it will also be important to further reveal the roles of Baicalein in ROS, interleukins, or lipopolysaccharide-induced NF-κB activation.

Eosinophils play a key role in the development of allergic inflammation including airway remodeling. A growing number of studies have demonstrated that a lack of eosinophils reduces airway mucus secretion, AHR, collagen deposition, and airway smooth muscle hypertrophy [42]. Eosinophils migrate to the airway in response to specific cytokines, such as IL-4, IL-5, IL-9, and IL-13 [43]. During allergic inflammation, eosinophils interact with airway epithelial cells to stimulate the NF-κB-dependent production of cytokines and adhesion molecules [38]. Additionally, the NF-κB pathway is important in eosinophil activation and survival [44]. In recent years, more attention has been paid to objective measures to guide the diagnosis and management of allergic eosinophilic airway inflammation. Fractional exhaled nitric oxide (FeNO) refers to the amount of NO measured when a person exhales, and is regarded as a new strategy to assess eosinophilic airway inflammation [45]. Numerous studies also provide evidence of increased FeNO following allergen provocation of allergic asthmatics [46, 47]. NO is an important endogenous modulator of airway and distal lung constriction, and the synthesis of NO in the airway is catalyzed by the activity of iNOS [48]. The expression of iNOS is increased in bronchial epithelial cells of patients with asthma and is correlated with the exhalation of NO [49]. In this study, treatment with Baicalein markedly decreased iNOS expression in allergic mouse lung tissue. The proinflammatory cytokines IL-4, IL-5 and IL-13 might be able to upregulate the generation of iNOS-derived NO through activating the NF-κB pathway [50]. Therefore, it is reasonable to assume that a reduction in iNOS expression is inseparable from the direct inhibition of Th2 cytokine release and NF-κB pathway activation.

Airway mucus hypersecretion, a hallmark of asthma pathogenesis, has long been recognized as an important cause of death in asthma. To date, more than 20 human mucin genes have been identified, and the principal airway gel-forming mucins in asthma are MUC5AC and MUC5B [51]. In our study, MUC5AC and MUC5B mRNA levels were significantly increased in asthma mice compared to those in control mice, and Baicalein treatment induced substantial decreases in MUC5AC and MUC5B mRNA expression levels. MUC5AC is mainly expressed in the epithelium, and significantly increased levels of MUC5AC are required for airflow obstruction in murine asthma models. MUC5B is expressed mainly in submucosal glands, and while the level of MUC5B differs from that of MUC5AC, MUC5B expression remains controversial. The expression of MUC5B was elevated in OVA-induced mouse asthmatic airways, and large amounts of glandular MUC5B extracellular mucus were observed in patients with mild asthma [52–54]. However, in recent years, MUC5B has been shown to have physiologic functions in the mucus that ensure its normal clearance, and the levels of MUC5B in asthma remain stable or even decrease in some cases [55]. Because IL-13 induced goblet cell hyperplasia and mucus hypersecretion in a murine asthmatic model and human airway epithelial through increasing MUC5AC expression, while the effect of IL-13 on MUC5B was more variable, we believe that the differences between the levels of MUC5AC and MUC5B are associated with IL-13. For example, IL-13 induces MUC5B expression in mouse models but, in contrast, frequently decreases MUC5B expression in cultured human airway epithelial cells; this difference in MUC5B expression may reflect the inter-species differences [56]. Due to the unstable expression of MUC5B, its contribution to mucus dysfunction in asthma requires further exploration.

Increased ECM deposition is another structural alteration described in asthma. Myofibroblasts and fibroblasts are the main producers of ECM components in the lung. Myofibroblasts deposit collagen types I and III during allergic airway inflammation. Fibroblasts secrete MMPs that are responsible for breaking down and regulating the components of the ECM, particularly collagens [57]. MMP-9, the dominant airway MMP, is up-regulated in allergic asthma, which causes airway remodeling [58]. The data presented herein suggest that Baicalein administration decreases the OVA-induced expression of collagen I and MMP-9. Chronic inflammation may drive airway remodeling, but this standpoint has been increasingly disputed. Apparent structural airway changes can be seen in patients with even mild asthma [28]. In our murine asthmatic model, a certain degree of airway remodeling was observed with OVA challenge for only 7 times, which is similar to the methods used in Gao’s and Yao’s studies [59, 60]. Inflammation and remodeling may have occurred in parallel instead of sequentially [28]. Our results demonstrate that Baicalein relieved this remodeling.

## CONCLUSIONS

In summary, our results demonstrate that Baicalein, a natural product from the traditional Chinese medication Huang Qin, effectively decreases OVA-induced eosinophilic airway inflammation, mucus overproduction, airway remodeling and AHR, most likely through inactivating the NF-κB pathway. Our findings provide evidence suggesting that Baicalein as a preventative or therapeutic drug for the treatment of asthma.

## MATERIALS AND METHODS

### Cells culture

BEAS-2B cells were purchased from the Shanghai Institute of Biochemistry and Cell Biology (Shanghai, People’s Republic of China) and were cultured in RPMI 1640 medium (Gibco, Eggenstein, Germany). BEAS-2B cell culture medium was supplemented with 10% heat-inactivated fetal bovine serum (FBS, HyClone, Logan, UT, USA), 100 U/mL penicillin, and 100 mg/mL streptomycin.

### Experimental animals and ethics approval

Female wild-type (WT) C57BL/6 mice that were 8–10 weeks old were obtained from the Wenzhou Medical University Animal Center. Animals were housed at constant room temperature with a 12-h day/night cycle and fed a standard rodent diet and water. All animal care and experimental procedures were approved by the Wenzhou Medical University Animal Policy and Welfare Committee (Approval Document No. wydw2016-0124).

### Model of OVA-induced airway inflammation and AHR

The mice were randomly assigned to four groups of 6 mice each: a control group (CON), an OVA group (OVA/Vehicle), a low dose Baicalein (10 mg/kg, Aladdin, China) treatment group (OVA+BAI 10) and a high dose Baicalein (20 mg/kg) treatment group (OVA+BAI 20). On days 0 and 14 of the experiment, mice in the OVA/Vehicle and Baicalein treatment groups (both low dose and high dose Baicalein) were sensitized by the intraperitoneal (i.p.) injection of OVA (20 μg, Sigma-Aldrich, Co., St Louis, USA) and Al(OH)_3_ (2 mg, Sigma-Aldrich, Co., St Louis, USA) suspended in 0.2 mL of saline. On days 25–31, mice in the OVA/Vehicle and Baicalein treatment groups were challenged with 1% OVA aerosol for 40 min each day to construct an asthmatic mouse model. The mice in the control group were administered normal saline with Al(OH)_3_ i.p. on days 0 and 14 of the experiment and were exposed to aerosolized saline for 40 min per day between days 25–31. Beginning on day 25 of the experiment, the mice were administered with 10 mg/kg or 20 mg/kg Baicalein (in 0.4% sodium carboxymethyl cellulose solution) or vehicle by gavage for 12 h each day (Figure 1A). Lung mechanics together with collection of serum, tissues and BALF were assessed at 24 h after the last challenge (day 31). Serum was used for IgE measurement using ELISA kits. Th2 cytokines and cellular measurements were assessed in BALF samples. The middle lobes of the right lungs were fixed in formalin and embedded in paraffin for histological analysis. The remaining lung tissues were used for RNA isolation and protein lysate preparation.

### Measurement of AHR

Following the final OVA challenge, the mice were anesthetized (80 mg/kg pentobarbital-NA, i.p.), tracheostomized (18-gauge cannula) for mechanical ventilation, and then connected to a computer-controlled small-animal mechanical ventilator (flexiVent; SCIREQ) to assess lung function as previously described [61]. Mice were mechanically ventilated at 200 breaths/min with a tidal volume of 0.25 mL and a positive end-expiratory pressure of 3 cm H_2_O (to mimic spontaneous ventilation). After baseline measurement, the mice were challenged for 10 s with saline aerosol and increasing concentrations (3.125–50 mg/mL) of Mch (Sigma-Aldrich, Co., St Louis, USA) at 4 to 5 min intervals. The peak response to each Mch dose was calculated as the mean of the three maximal values and was used to calculate airway dynamic compliance.

### BALF collection

The chest cavity of each mouse was carefully opened, followed by the ligation of the left lung. The left lung was infused thrice with 1 mL PBS to obtain BALF as previously described [62]. The collected BALF was centrifuged for 10 min at 1,000 rpm. Target cytokines were the measured in the cell-free supernatant. The cell pellets from the BALF were rinsed and resuspended in 50 μL of PBS. The total number of cells in the BALF was detected by using a cell counting instrument. The number of eosinophils in the BALF was determined using Wright-Giemsa staining, with at least 200 cells counted per slide.

### ELISA

The levels of IL-4, IL-5, and IL-13 in the BALF supernatant and lung homogenates were detected using ELISA kits (eBioscience, San Diego, CA, USA) according to the manufacturer’s instructions (Minneapolis, MN). Briefly, after blocking the plate, 100 μL of BALF supernatant and lung homogenates were added to an ELISA plate coated with monoclonal capture antibodies and incubated at room temperature for 2 h. Then, the plate was washed 5 times with PBST (PBS solution containing 0.5% Tween-20) and monoclonal detection antibodies conjugated with horseradish peroxidase were added. After incubation at room temperature for 1 h, the plate was washed and supplemented with tetramethylbenzidine. The reaction was stopped by the addition of 2 N H_2_SO_4_. The absorbance at a wavelength of 450 nm was measured using a SpectraMax M5 plate reader (Molecular Devices, Sunnyvale, CA). A standard curve was drawn using purified proteins supplied with the ELISA kit.

### Histopathological study

The middle lobe of the right lung was collected and fixed in 4% paraformaldehyde, embedded in paraffin and cut into 5-micron sections. The sections were stained with H&E, PAS, and Sirius Red in accordance with the standard light microscopy protocol. The H&E stained sections were scored blindly for the severity of inflammatory cell infiltration, and peribronchial cell counts were performed blind based on the following 5-point scoring system: 0, no cells; 1, a few cells; 2, a ring of cells in a layer one cell deep; 3, a ring of cells two to four cells deep; 4, a ring of cells of more than four cells deep. To quantify mucus production in the lung, PAS sections were randomized, examined in a blinded fashion and scored on a scale from 0 to 4 as follows: 0, no goblet cells, 1, <25% goblet cells; 2, 25–50% goblet cells; 3: 50–75% goblet cells; 4, >75% goblet cells. Inflammatory cells and goblet cells were scored in at least three different fields for each lung section. Mean scores were obtained.

To quantify the extent of fibrosis, the percentage of fibrosis indicated by Sirius Red staining in ten representative images taken from each lung section was determined by Image-Pro Plus (Media Cybernetics Inc., Silver Spring, MD).

All histopathological evaluations were performed in duplicate by a blinded independent observer.

### Assay of cellular NF-κB p65 translocation

BEAS-2B cells and lung sections were immunofluorescence-labeled using a cellular NF-κB p65 Translocation Kit (Beyotime Biotech, Nantong, Jiangsu, China) according to the manufacturer’s instruction. The p65 protein and nuclear fluorescence are shown in red and blue, respectively, and were simultaneously viewed with a fluorescence microscope (200×, Nikon, Tokyo, Japan) at an excitation wavelength of 350 nm for 4′,6-diamidino-2-phenylindole·2HCl (DAPI) stained cells and 540 nm for cyanine 3 (Cy3)-stained cells. The red and blue images were overlaid to create a two-color image. Quantitative analysis of nuclear p65 fluorescence intensity in four representative images were analyzed by Image J software.

### Western blot analysis

BEAS-2B cells were treated with Baicalein (2.5 μM) or vehicle (DMSO) for 30 min, followed by TNF-α (10 ng/mL) exposure for 60 min and 2 h to collect total protein and nuclear and cytosolic proteins, respectively. Lung (100 μg) and cellular (50 μg) protein samples were subjected to 10% sodium dodecyl sulfate polyacrylamide gel electrophoresis (SDS-PAGE) and transferred onto a polyvinylidene fluoride (PVDF) membrane (Bio-Rad Laboratories Inc, USA). After blocking in blocking buffer (5% milk in Tris-buffered saline containing 0.05% Tween 20 [TBST]) for 1.5 h at room temperature, the membranes were incubated with different primary antibodies overnight at 4 °C. Afterwards, the membranes were washed in TBST and reacted with secondary horseradish peroxidase-conjugated antibody (Santa Cruz, CA, USA; 1:3000) for 1–2 h at room temperature. Blots were then visualized using enhanced chemiluminescence reagents (Bio-Rad Laboratories Inc, USA). The densities of the immunoreactive bands were analyzed using ImageJ software (NIH, Bethesda, MD, USA). Antibodies against IκBα (1:300), NF-κB p65 subunit (1:300), and lamin B (1:300) were purchased from Santa Cruz Technology (Santa Cruz, CA, USA). Antibodies against P-IκBα (1:1,000) and inducible nitric oxide synthase (iNOS, 1:1,000) were purchased from Cell Signaling Technology (Danvers, MA, USA).

### Isolation of nuclear and cytoplasmic proteins

Nuclear proteins were prepared using a cytoplasmic and nuclear protein extraction kit (KeyGEN, Nanjing, China). Briefly, BEAS-2B cells and lung tissues were incubated in 10 volumes of hypotonic buffer A (20 mM HEPES, pH 7.9, 1.5 mM MgCl_2_, and 10 mM KCl) and one tenth buffer B on ice for 15 min and homogenized. Nuclei were recovered by centrifugation at 16,000×g for 5 min, and the supernatant was collected as the cytosolic extracts. The nuclei were extracted in buffer C (20 mM HEPES, pH 7.9, 25% glycerol, 420 mM NaCl, 0.2 mM EDTA, and 1.5 mM MgCl_2_) 4 times (for 10 min each time) on ice. Insoluble material was removed by centrifugation at 16 000×g for 10 min, and the supernatant was used as the nuclear extract.

### Real-time quantitative polymerase chain reaction (RT-qPCR)

Total RNA was isolated from lung tissues using TRIzol-reagent and quantified by ultraviolet (UV) absorption at 260 and 280 nm. Both reverse transcription and qPCR were performed using a two-step M-MLV Platinum SYBR Green qPCR SuperMix-UDG kit. An Eppendorf Master cycler ep RealPlex detection system (Eppendorf, Hamburg, Germany) was used for RT-qPCR analysis. Primers complementary to the genes encoding IL-4, IL-5, IL-13, Muc5AC, Muc5B, MMP-9, collagen I and β-actin, were synthesized by Invitrogen (Shanghai, China), and their sequences are presented in Supplementary Table 1. The expression of each gene was determined and normalized to the expression of β-actin.

### iNOS activity assay

The total NO synthase (TNOS) and iNOS activity levels in the serum were determined by using an NOS Assay Kit (Nanjing Jiancheng, Nanjing, China) following the manufacturer’s protocol.

### Statistical analysis

Data were analyzed using GraphPad Prism 6.0 software. Values are expressed as the mean ± standard error of measurement (SEM). One-way analysis of variance (ANOVA) followed by Dunnett’s post hoc test was employed to analyze the differences between sets of data. A p-value less than 0.05 indicated statistical significance and is denoted as *or ^#^. *In vitro* experiments were performed with n ≥ 3 independent repeats. *In vivo* experiments were performed with n ≥ 6 mice in each group.

## Supplementary Material

Supplementary Figures

Supplementary Table 1
