# Supplementary material for: Baicalein attenuates OVA-induced allergic airway inflammation through the inhibition of the NF-κB signaling pathway
Source: Aging (Albany NY). 2019 Nov 6;11(21):9310–27. doi: 10.18632/aging.102371 (PMC6874438; doi:10.18632/aging.102371)
Supplement: Supplementary Figures [file aging-11-102371-s002.pdf]

SUPPLEMENTARY FIGURES

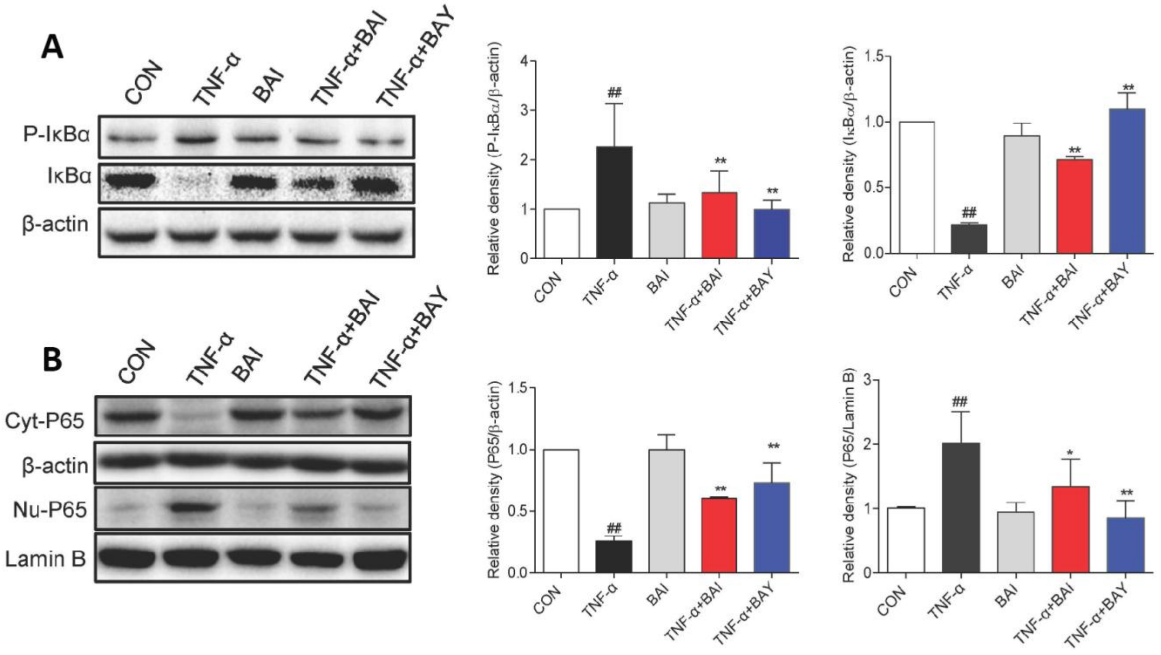

Supplementary Figure 1. The effects of Baicalein and NF-κB inhibitor BAY11-7082 on TNF-α-induced NF-κB activation in BEAS-2B cells. (**A**) BEAS-2B cells were pre-treated with Baicalein (2.5 μM) and BAY11-7082 (2.5 μM) or vehicle for 30 min and then stimulated with TNF-α (10 ng/mL) for 60 min. Total protein were extracted and analyzed for P-IκBα and IκBα expression by western blot analysis, with β-actin used as the internal control. (**B**) After BEAS-2B cells were exposed to TNF-α (10 ng/mL) for 2 h, the nuclear and cytosolic proteins were separated using cytoplasmic and nuclear protein extraction kit, nuclear and cytosolic p65 levels were determined by western blot analysis. β-actin and Lamin B were used as the internal controls, respectively. (mean ± SEM of more than three independent experiments; $^{\#\#}$*P<0.01*, vs CON group; **P*<0.05, ***P*<0.01, vs TNF-α group).

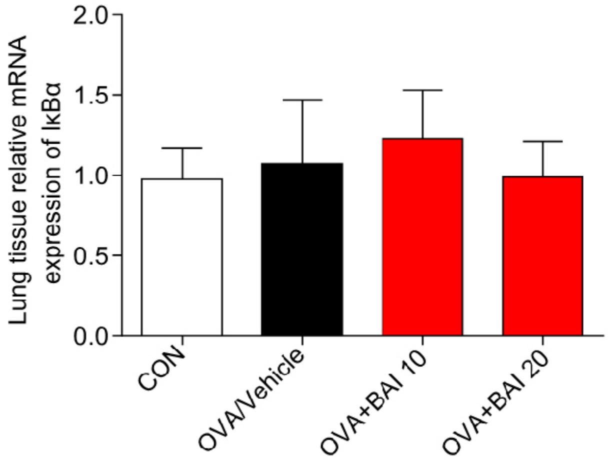

Supplementary Figure 2. The effects of Baicalein on IκB mRNA expression in vivo. 24 h after the last challenge, lungs were isolated and gene expression levels of IκBα were determined in lung tissues by RT-qPCR. Data were normalized to β-actin (Results are presented as the mean ± SEM; n = 6 mice per group).
