# Supplementary material for: Baicalein attenuates OVA-induced allergic airway inflammation through the inhibition of the NF-κB signaling pathway
Source: Aging (Albany NY). 2019 Nov 6;11(21):9310–27. doi: 10.18632/aging.102371 (PMC6874438; doi:10.18632/aging.102371)
Supplement: Supplementary Table 1 [file aging-11-102371-s001.pdf]

SUPPLEMENTARY TABLE

Supplementary Table 1. Primers used for real-time qPCR assay.

Gene	Species	FW	RW
IL-4	Mouse	GGAACAAAGACCTGTGGGGT	GGCATCGAAAAGCCCGAAAG
IL-5	Mouse	TCCTCTTCGTTGCATCAGGG	TGTGGCTGGCTCTCATTCAC
IL-13	Mouse	TGCCATCTACAGGACCCAGA	CTCATTAGAAGGGGCCGTGG
MUC5AC	Mouse	TCTACTGACTGCACCAACACA	CCACACTTTCGCAGCTCAAC
MUC5B	Mouse	GATTCGGCCGAGGCAAGTA	GAGGCCAAAACAGCCAACAG
MMP9	Mouse	TCTTCTGGCGTGTGAGTTTCC	CGGTTGAAGCAAAGAAGGAGC
Collagen I	Mouse	TGGCCTTGGAGGAAACTTTG	CTTGGAAACCTTGTGGACCAG
IκBα	Mouse	TGAAGGACGAGGAGTACGAGC	TGCAGGAACGAGTCTCCGT
β-actin	Mouse	CCGTGAAAAGATGACCCAGA	TACGACCAGAGGCATACAG
IκBα	Human	ACCTGGTGTCACTCCTGTTGA	CTGCTGCTGTATCCGGGTG
β-actin	Human	CCTGGCACCCAGCACAAT	GCCGATCCACACGGAGTACT
